# *tert*-Butyl 2-(3-acetyl­amino-2-oxo-1,2-dihydro-1-pyrid­yl)acetate

**DOI:** 10.1107/S1600536808039810

**Published:** 2008-11-29

**Authors:** N. David Karis, Wendy A. Loughlin, Ian D. Jenkins, Peter C. Healy

**Affiliations:** a Eskitis Institute for Cell and Molecular Therapies, Griffith University, Nathan, Brisbane 4111, Australia; b School of Biomolecular and Physical Sciences and Eskitis Institute for Cell and Molecular Therapies, Griffith University, Nathan, Brisbane 4111, Australia

## Abstract

The title compound, C_13_H_18_N_2_O_4_, crystallizes as discrete mol­ecules associated as N—H⋯O hydrogen-bonded dimers disposed about a crystallographic inversion centre. The structure is the first solid-state structure for a 3-acetyl­pyridone without C-4 to C-6 substituents. The amide subsituent at C-3 is coplanar with the pyridone ring, while the *tert*-butyl ester group is orthogonal to the pyridine ring. The amide and ester carbonyl O atoms are not involved in strong hydrogen bonding with only a number of intramolecular and intermolecular C—H⋯O inter­actions apparent in the structure.

## Related literature

For general background, see: Bernstein *et al.* (1994 ▶); Dragovich *et al.* (2002 ▶); Hu *et al.* (2008 ▶); Karis *et al.* (2007 ▶); Kim *et al.* (2008 ▶); Loughlin *et al.* (2004 ▶); Reiner *et al.* (1999 ▶); Semple *et al.* (1998 ▶); Veale *et al.* (1995 ▶). For the synthesis, see: Sanderson *et al.* (1997 ▶); Tamura *et al.* (1996 ▶). For related structures. see: Karis *et al.* (2006 ▶); Yang & Craven (1998 ▶).
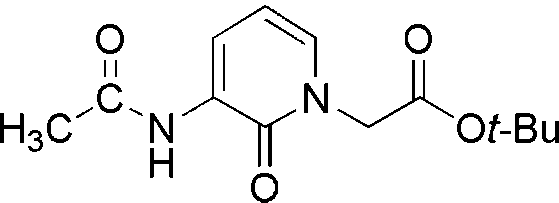


## Experimental

### 

#### Crystal data

C_13_H_18_N_2_O_4_
*M* *_r_* = 266.29
Monoclinic, 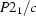

*a* = 13.9417 (15) Å
*b* = 5.585 (1) Å
*c* = 17.861 (2) Å
β = 97.039 (9)°
*V* = 1380.3 (3) Å^3^
*Z* = 4
Mo *K*α radiation
μ = 0.10 mm^−1^
*T* = 295 (2) K
0.40 × 0.30 × 0.20 mm

#### Data collection

Rigaku AFC-7R diffractometer
Absorption correction: none
2731 measured reflections
2428 independent reflections
1482 reflections with *I* > 2σ(*I*)
*R*_int_ = 0.046
3 standard reflections every 150 reflections intensity decay: 0.6%

#### Refinement

*R*[*F* ^2^ > 2σ(*F* ^2^)] = 0.052
*wR*(*F* ^2^) = 0.162
*S* = 1.02
2428 reflections
176 parameters
H-atom parameters constrained
Δρ_max_ = 0.27 e Å^−3^
Δρ_min_ = −0.27 e Å^−3^

### 

Data collection: *MSC/AFC7 Diffractometer Control Software* (Molecular Structure Corporation, 1999 ▶); cell refinement: *MSC/AFC7 Diffractometer Control Software*; data reduction: *TEXSAN for Windows* (Molecular Structure Corporation, 2001 ▶); program(s) used to solve structure: *TEXSAN for Windows*; program(s) used to refine structure: *TEXSAN for Windows* and *SHELXL97* (Sheldrick, 2008 ▶); molecular graphics: *ORTEP-3* (Farrugia, 1997 ▶); software used to prepare material for publication: *TEXSAN for Windows* and *PLATON* (Spek, 2003 ▶).

## Supplementary Material

Crystal structure: contains datablocks global, I. DOI: 10.1107/S1600536808039810/bt2816sup1.cif

Structure factors: contains datablocks I. DOI: 10.1107/S1600536808039810/bt2816Isup2.hkl

Additional supplementary materials:  crystallographic information; 3D view; checkCIF report

## Figures and Tables

**Table 1:** Hydrogen-bond geometry (Å, °)

*D*—H⋯*A*	*D*—H	H⋯*A*	*D*⋯*A*	*D*—H⋯*A*
N3—H3⋯O2^i^	0.86	2.34	3.164 (3)	161
C4—H4⋯O3	0.95	2.23	2.830 (3)	120
C14—H14*A*⋯O3^ii^	0.96	2.56	3.465 (4)	157
C15—H15*A*⋯O11	0.96	2.44	2.980 (4)	115
C16—H16*A*⋯O11	0.96	2.44	2.978 (5)	115
C32—H32*C*⋯O2^i^	0.96	2.33	3.222 (3)	155

Symmetry codes: (i) 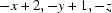; (ii) 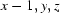.

**Fig. 1.**
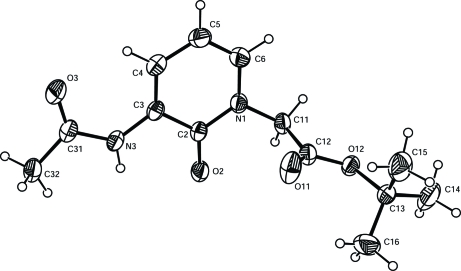
View of the molecular structure of (II) with the atom numbering scheme. Displacement ellipsoids for non-H atoms are drawn at the 30% probability level. H atoms are presented as small spheres of arbitrary radii.

**Fig. 2.**
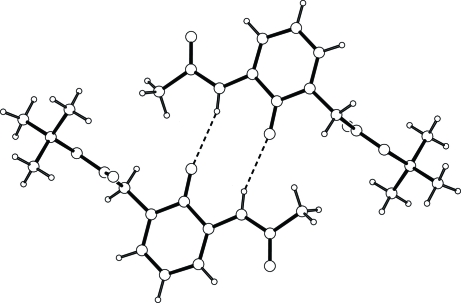
View of the dimeric structure of (II).

**Fig. 3.**
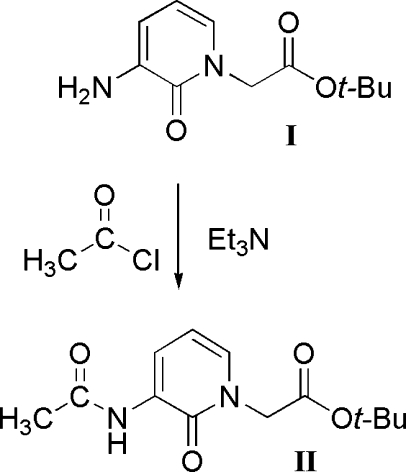
The formation of the title compound.

## supplementary crystallographic information

### Comment

Increased binding affinity of pyridone based scaffolds as P4—P2 conformational restraints (Dragovich *et al.*, 2002; Reiner *et al.*, 1999; Semple *et al.*, 1998; Veale *et al.*, 1995; Bernstein *et al.*, 1994) in peptidiomimetics (Loughlin *et al.*, 2004, and references therein) is often associated with the substituent functionality at N1 and C3 of the pyridone ring. This has been reflected by enzyme-ligand crystal structures of a C3-amidoaryl pyridone with human rhinovirus (HRV) 3 C protease (3CP) (Dragovich *et al.*, 2002), and a C3-sulfonylamide pyridone with porcine pancreatic elastase (Bernstein *et al.*, 1994). Similiarly, other enzyme-ligand interactions have been observed in the solid state with kinases; a N1-aryl pyridone with Met kinase (Kim *et al.*, 2008) and a N1-aryl C3-aryl pyridone with KDR kinase (Hu *et al.*, 2008). Thus an understanding of the structure of substituted pyridone compounds is important. Elsewhere, the facile synthesis of N1, C3-substituted pyridones is reported (Karis *et al.*, 2007). Herein we report the first solid state structure (II) for a 3-acetylpyridone without C4 to C6 substituents.

The structure of (II) consists of discrete molecular units (Fig. 1) which form N3—H3···O2 hydrogen bonded dimers disposed about a crystallographic centre of symmetry (Figure 2, Table 1). The amide N3—C31—O3—C32 is co-planar with the pyridone ring with the O3···H4 contact distance 2.23 Å. The *tert*-butyl ester group attached to N1 lies orthogonal to the pyridone ring with the C2—N1—C11—C12 torsion angle -81.1 (2)°. The geometry of the pyridone ring is in accord with related structures (Yang & Craven, 1998; Karis *et al.*, 2006) with the C2—C3 distance 1.440 (3)Å while the other C—C and C—N distance range from 1.333 (4) - 1.402 (4) Å. The N3—C31 distance of 1.362 (3)Å is shorter than the N3—C3 distance of 1.399 (3) Å; indicating a preference for involvement of N3 in conjugation with the amide rather than the pyridone. The carbonyl groups C31—O3 and C21—O11 are not involved in strong hydrogen bonding interactions with only a number of C—H···O interactions apparent in the crystal lattice (Table 2).

### Experimental

Tert-Butyl 2-(3'-amino-2'-oxopyridin-1'(2*H*)-yl)acetate (compound (I)) was prepared by *N*-alkylation of nitropyridone with sodium hydride and *tert*-butyl bromoacetate (Sanderson *et al.*, 1997; Tamura *et al.*, 1996), and subsequent hydrogenation over palladium-on-carbon (Tamura *et al.*, 1996).

For the preparation of compound (II), compound (I) (0.78 g, 3.48 mmol) was dissolved in a mixture of dry dichloromethane (10 ml) and triethylamine (0.97 ml, 6.96 mmol) under nitrogen. Acetyl chloride (0.50 ml, 6.96 mmol) was added dropwise at 295 K. The resulting mixture was stirred for 4 h and then concentrated to give a suspension of the product and triethylamine hydrochloride. The suspension was directly transferred to a silica gel column using dichloromethane with 0.5% triethylamine and eluted with an ethyl acetate /dichloromethane gradient (0 to 20% ethyl acetate, with 0.5% triethyl amine. Red crystals of (II) (m.p. 415–418 K) (0.91 g, 98%) were isolated by slow evaporation from an ethyl acetate /dichloromethane solution. Analysis found: C 58.73, H 6.84, N 10.36%; calculated for C_13_H_18_N_2_O_4_: C 58.64, H 6.81, N 10.52%. ν_max_(KBr) cm^-1^ 3318, 2974, 1716, 1646, 1605, 1532, 1512. δ_H_ (400 MHz, CDCl_3_, p.p.m.) 1.49 (9*H*, s, C(CH_3_)_3_), 2.19 (3*H*, s, CH_3_), 4.58 (2*H*, s, H2), 6.27 (1*H*, dd, J = 7.0, 7.0 Hz, H5'), 6.92 (1*H*, dd, J = 7.0, 1.6 Hz, H6'), 8.35 (1*H*, brs, W_h1/2_ = 11 Hz, NH), 8.39 (1*H*, dd, J = 7.2, 1.6 Hz, H4'). δ_C_ (100 MHz, CDCl_3_) 24.7 (CH_3_), 28.0 (C(CH_3_)_3_), 51.6 (C2), 83.1 (C(CH_3_)_3_), 106.8 (C5'), 122.4 (C4'), 129.3 (C3'), 130.2 (C6'), 157.4 (C2'), 166.2 (C1), 169.0 (CO). MS (ES^+^) 289.2 (MNa^+^, 30%) 273.2 (MLi^+^, 40%).

### Refinement

Carbon bonded H atoms were included in idealized positions and refined as riding atoms, with C—H set to 0.95–0.96 Å. *U*_iso_(H) values were set to 1.2*U*_eq_ (aromatic, methylene) and 1.5*U*_eq_ (methyl) of the parent atom. The amide proton was located from difference Fourier maps and refined with N—H set to 0.86Å and *U*_iso_(H) values set to 1.2*U*_eq_ of the parent atom. Considerable thermal motion was apparent in the peripheral carbons of the *tert*-butyl group.

### Crystal data

**Table:**

C_13_H_18_N_2_O_4_	*F*_000_ = 568
*M**_r_* = 266.29	*D*_x_ = 1.281 Mg m^−^^3^
Monoclinic, *P*2_1_/*c*	Mo *K*α radiation λ = 0.71069 Å
Hall symbol: -P 2ybc	Cell parameters from 25 reflections
*a* = 13.9417 (15) Å	θ = 12.6–17.5º
*b* = 5.585 (1) Å	µ = 0.10 mm^−^^1^
*c* = 17.861 (2) Å	*T* = 295 (2) K
β = 97.039 (9)º	Block, red
*V* = 1380.3 (3) Å^3^	0.40 × 0.30 × 0.20 mm
*Z* = 4	

### Data collection

**Table:**

Rigaku AFC-7R diffractometer	*R*_int_ = 0.046
Radiation source: Rigaku rotating anode	θ_max_ = 25.0º
Monochromator: graphite	θ_min_ = 2.6º
*T* = 295 K	*h* = −16→16
ω–2θ scans	*k* = −6→0
Absorption correction: none	*l* = −21→10
2731 measured reflections	3 standard reflections
2428 independent reflections	every 150 reflections
1482 reflections with *I* > 2σ(*I*)	intensity decay: 0.6%

### Refinement

**Table:**

Refinement on *F*^2^	Secondary atom site location: difference Fourier map
Least-squares matrix: full	Hydrogen site location: inferred from neighbouring sites
*R*[*F*^2^ > 2σ(*F*^2^)] = 0.052	H-atom parameters constrained
*wR*(*F*^2^) = 0.162	*w* = 1/[σ^2^(*F*_o_^2^) + (0.0906*P*)^2^ + 0.0473*P*] where *P* = (*F*_o_^2^ + 2*F*_c_^2^)/3
*S* = 1.02	(Δ/σ)_max_ = 0.001
2428 reflections	Δρ_max_ = 0.27 e Å^−^^3^
176 parameters	Δρ_min_ = −0.27 e Å^−^^3^
Primary atom site location: structure-invariant direct methods	Extinction correction: none

### Special details

**Table:**

Experimental. The scan width was (1.79 + 0.30tanθ)° with an ω scan speed of 16° per minute (up to 4 scans to achieve I/σ(I) > 10). Stationary background counts were recorded at each end of the scan, and the scan time:background time ratio was 2:1.
Geometry. Bond distances, angles *etc*. have been calculated using the rounded fractional coordinates. All su's are estimated from the variances of the (full) variance-covariance matrix. The cell e.s.d.'s are taken into account in the estimation of distances, angles and torsion angles
Refinement. Refinement on *F*^2^ for ALL reflections except those flagged by the user for potential systematic errors. Weighted *R*-factors *wR* and all goodnesses of fit *S* are based on *F*^2^, conventional *R*-factors *R* are based on *F*, with *F* set to zero for negative *F*^2^. The observed criterion of *F*^2^ > σ(*F*^2^) is used only for calculating -*R*-factor-obs *etc*. and is not relevant to the choice of reflections for refinement. *R*-factors based on *F*^2^ are statistically about twice as large as those based on *F*, and *R*-factors based on ALL data will be even larger.

### Fractional atomic coordinates and isotropic or equivalent isotropic displacement parameters (Å^2^)

**Table:**

	*x*	*y*	*z*	*U*_iso_*/*U*_eq_	
O2	0.93631 (11)	0.2790 (3)	0.03272 (10)	0.0595 (6)	
O3	1.26910 (13)	0.2449 (4)	0.15628 (14)	0.0940 (9)	
O11	0.79117 (14)	0.2673 (4)	0.16249 (13)	0.0868 (9)	
O12	0.66171 (11)	0.0894 (3)	0.09995 (9)	0.0520 (6)	
N1	0.91347 (13)	−0.0488 (3)	0.10449 (11)	0.0487 (6)	
N3	1.12376 (12)	0.3204 (4)	0.08928 (11)	0.0497 (7)	
C2	0.97001 (15)	0.1321 (4)	0.08098 (12)	0.0450 (7)	
C3	1.06936 (15)	0.1361 (4)	0.11623 (12)	0.0450 (7)	
C4	1.10110 (17)	−0.0279 (5)	0.16998 (14)	0.0546 (8)	
C5	1.0390 (2)	−0.2076 (5)	0.19027 (15)	0.0634 (10)	
C6	0.94768 (18)	−0.2158 (5)	0.15792 (15)	0.0580 (9)	
C11	0.81146 (16)	−0.0501 (4)	0.07545 (14)	0.0508 (8)	
C12	0.75508 (16)	0.1222 (4)	0.11859 (14)	0.0505 (8)	
C13	0.58947 (17)	0.2243 (5)	0.13832 (15)	0.0569 (9)	
C14	0.4945 (2)	0.1252 (7)	0.1020 (2)	0.0982 (15)	
C15	0.6042 (2)	0.1659 (8)	0.22108 (18)	0.0934 (14)	
C16	0.5968 (3)	0.4858 (6)	0.1224 (3)	0.1150 (18)	
C31	1.21909 (17)	0.3673 (5)	0.11050 (15)	0.0577 (9)	
C32	1.25913 (17)	0.5768 (6)	0.07310 (16)	0.0683 (10)	
H3	1.09400	0.41360	0.05580	0.0590*	
H4	1.16570	−0.02040	0.19410	0.0650*	
H5	1.06240	−0.32280	0.22700	0.0750*	
H6	0.90590	−0.33820	0.17180	0.0690*	
H14A	0.44270	0.19330	0.12570	0.1480*	
H14B	0.49430	−0.04570	0.10780	0.1480*	
H14C	0.48610	0.16470	0.04930	0.1480*	
H15A	0.66300	0.23860	0.24400	0.1400*	
H15B	0.60820	−0.00460	0.22760	0.1400*	
H15C	0.55080	0.22640	0.24460	0.1400*	
H16A	0.65600	0.54750	0.14840	0.1730*	
H16B	0.54300	0.56810	0.13940	0.1730*	
H16C	0.59610	0.50990	0.06910	0.1730*	
H32A	1.31990	0.53370	0.05670	0.1030*	
H32B	1.26860	0.70740	0.10820	0.1030*	
H32C	1.21470	0.62420	0.03030	0.1030*	
H111	0.78650	−0.20760	0.07980	0.0610*	
H112	0.80430	−0.00530	0.02370	0.0610*	

### Atomic displacement parameters (Å^2^)

**Table:**

	*U*^11^	*U*^22^	*U*^33^	*U*^12^	*U*^13^	*U*^23^
O2	0.0418 (9)	0.0716 (12)	0.0638 (10)	−0.0042 (9)	0.0008 (8)	0.0138 (10)
O3	0.0447 (10)	0.1068 (17)	0.1239 (19)	−0.0035 (11)	−0.0167 (11)	0.0397 (15)
O11	0.0571 (12)	0.0988 (16)	0.1066 (16)	−0.0158 (11)	0.0190 (11)	−0.0554 (14)
O12	0.0417 (9)	0.0507 (10)	0.0646 (10)	0.0020 (7)	0.0110 (7)	−0.0070 (9)
N1	0.0405 (10)	0.0493 (11)	0.0577 (12)	−0.0012 (9)	0.0119 (9)	−0.0033 (10)
N3	0.0332 (10)	0.0621 (13)	0.0531 (11)	0.0032 (9)	0.0028 (8)	0.0019 (10)
C2	0.0375 (11)	0.0517 (14)	0.0468 (12)	0.0008 (11)	0.0096 (10)	−0.0036 (12)
C3	0.0375 (11)	0.0528 (14)	0.0461 (12)	0.0056 (10)	0.0111 (10)	−0.0059 (11)
C4	0.0445 (13)	0.0635 (16)	0.0555 (14)	0.0082 (12)	0.0052 (11)	−0.0015 (13)
C5	0.0629 (16)	0.0598 (17)	0.0673 (17)	0.0109 (14)	0.0076 (13)	0.0107 (14)
C6	0.0583 (15)	0.0487 (15)	0.0680 (16)	0.0000 (12)	0.0118 (13)	0.0020 (13)
C11	0.0404 (12)	0.0517 (14)	0.0614 (14)	−0.0088 (11)	0.0112 (11)	−0.0087 (12)
C12	0.0421 (13)	0.0530 (14)	0.0575 (14)	−0.0060 (11)	0.0100 (11)	−0.0062 (12)
C13	0.0495 (14)	0.0502 (15)	0.0751 (17)	0.0081 (11)	0.0243 (12)	0.0020 (13)
C14	0.0472 (16)	0.105 (3)	0.142 (3)	0.0181 (17)	0.0101 (18)	−0.018 (2)
C15	0.083 (2)	0.122 (3)	0.083 (2)	0.014 (2)	0.0411 (18)	0.010 (2)
C16	0.127 (3)	0.0546 (19)	0.180 (4)	0.024 (2)	0.085 (3)	0.024 (2)
C31	0.0377 (13)	0.0680 (17)	0.0671 (16)	0.0021 (12)	0.0049 (12)	−0.0023 (14)
C32	0.0407 (13)	0.081 (2)	0.0817 (19)	−0.0074 (13)	0.0020 (13)	0.0100 (16)

### Geometric parameters (Å, °)

**Table:**

O2—C2	1.240 (3)	C31—C32	1.489 (4)
O3—C31	1.218 (3)	C4—H4	0.9500
O11—C12	1.195 (3)	C5—H5	0.9500
O12—C12	1.316 (3)	C6—H6	0.9500
O12—C13	1.490 (3)	C11—H111	0.9500
N1—C2	1.378 (3)	C11—H112	0.9500
N1—C6	1.377 (3)	C14—H14A	0.9600
N1—C11	1.453 (3)	C14—H14B	0.9600
N3—C3	1.399 (3)	C14—H14C	0.9600
N3—C31	1.362 (3)	C15—H15A	0.9600
N3—H3	0.8600	C15—H15B	0.9600
C2—C3	1.450 (3)	C15—H15C	0.9600
C3—C4	1.361 (3)	C16—H16A	0.9600
C4—C5	1.402 (4)	C16—H16B	0.9600
C5—C6	1.333 (4)	C16—H16C	0.9600
C11—C12	1.512 (3)	C32—H32A	0.9600
C13—C14	1.507 (4)	C32—H32B	0.9600
C13—C16	1.494 (4)	C32—H32C	0.9600
C13—C15	1.503 (4)		
			
O2···N3	2.694 (2)	C31···H4	2.7800
O2···C12	3.233 (3)	C32···H112^i^	3.0200
O2···C32^i^	3.222 (3)	H3···O2	2.3100
O2···N3^i^	3.164 (3)	H3···H32C	2.1500
O3···C4	2.830 (3)	H3···O2^i^	2.3400
O11···C2	3.130 (3)	H4···O3	2.2300
O11···N1	2.744 (3)	H4···C31	2.7800
O11···C15	2.980 (4)	H4···O11^vi^	2.8200
O11···C16	2.978 (5)	H5···O11^vi^	2.7100
O11···C5^ii^	3.319 (4)	H5···C5^vi^	3.0500
O11···C4^ii^	3.379 (3)	H5···C6^vi^	3.0200
O2···H3	2.3100	H6···O11^vii^	2.7200
O2···H32C^i^	2.3300	H6···H111	2.3100
O2···H112	2.4200	H6···C4^vi^	3.0300
O2···H3^i^	2.3400	H14A···O3^viii^	2.5600
O3···H4	2.2300	H14A···H15C	2.4600
O3···H14A^iii^	2.5600	H14A···H16B	2.5100
O3···H15B^ii^	2.8800	H14B···C16^vii^	2.9800
O11···H16A	2.4400	H14B···H15B	2.5100
O11···H6^iv^	2.7200	H14B···H16B^vii^	2.3100
O11···H15A	2.4400	H14C···H16C	2.4600
O11···H4^ii^	2.8200	H15A···O11	2.4400
O11···H5^ii^	2.7100	H15A···C12	2.7900
N1···O11	2.744 (3)	H15A···H16A	2.4200
N3···O2	2.694 (2)	H15B···C12	3.0800
N3···O2^i^	3.164 (3)	H15B···H14B	2.5100
N3···H112^v^	2.9400	H15B···O3^vi^	2.8800
C2···O11	3.130 (3)	H15C···H14A	2.4600
C2···C2^v^	3.441 (3)	H16A···O11	2.4400
C4···O3	2.830 (3)	H16A···C12	2.8300
C4···O11^vi^	3.379 (3)	H16A···H15A	2.4200
C5···O11^vi^	3.319 (4)	H16B···H14A	2.5100
C12···O2	3.233 (3)	H16B···H14B^iv^	2.3100
C15···O11	2.980 (4)	H16C···H14C	2.4600
C16···O11	2.978 (5)	H32B···C4^iv^	3.0800
C32···O2^i^	3.222 (3)	H32C···H3	2.1500
C4···H32B^vii^	3.0800	H32C···O2^i^	2.3300
C4···H6^ii^	3.0300	H32C···C11^i^	3.0300
C5···H5^ii^	3.0500	H32C···C12^i^	3.0900
C6···H5^ii^	3.0200	H32C···H112^i^	2.3400
C11···H32C^i^	3.0300	H111···H6	2.3100
C12···H15A	2.7900	H112···O2	2.4200
C12···H32C^i^	3.0900	H112···N3^v^	2.9400
C12···H16A	2.8300	H112···C32^i^	3.0200
C12···H15B	3.0800	H112···H32C^i^	2.3400
C16···H14B^iv^	2.9800		
			
C12—O12—C13	121.10 (18)	C6—C5—H5	120.00
C2—N1—C6	123.04 (19)	N1—C6—H6	120.00
C2—N1—C11	117.79 (18)	C5—C6—H6	120.00
C6—N1—C11	119.00 (19)	N1—C11—H111	109.00
C3—N3—C31	126.7 (2)	N1—C11—H112	109.00
C3—N3—H3	117.00	C12—C11—H111	109.00
C31—N3—H3	117.00	C12—C11—H112	109.00
N1—C2—C3	115.51 (19)	H111—C11—H112	109.00
O2—C2—N1	120.98 (19)	C13—C14—H14A	109.00
O2—C2—C3	123.5 (2)	C13—C14—H14B	109.00
N3—C3—C2	113.04 (19)	C13—C14—H14C	109.00
N3—C3—C4	126.5 (2)	H14A—C14—H14B	110.00
C2—C3—C4	120.5 (2)	H14A—C14—H14C	109.00
C3—C4—C5	120.4 (2)	H14B—C14—H14C	109.00
C4—C5—C6	120.0 (3)	C13—C15—H15A	110.00
N1—C6—C5	120.6 (2)	C13—C15—H15B	109.00
N1—C11—C12	111.23 (19)	C13—C15—H15C	110.00
O11—C12—C11	124.2 (2)	H15A—C15—H15B	109.00
O11—C12—O12	125.7 (2)	H15A—C15—H15C	109.00
O12—C12—C11	110.02 (19)	H15B—C15—H15C	109.00
O12—C13—C15	108.9 (2)	C13—C16—H16A	109.00
O12—C13—C14	103.0 (2)	C13—C16—H16B	109.00
C14—C13—C16	110.8 (3)	C13—C16—H16C	109.00
C15—C13—C16	113.3 (3)	H16A—C16—H16B	109.00
O12—C13—C16	109.9 (2)	H16A—C16—H16C	109.00
C14—C13—C15	110.5 (2)	H16B—C16—H16C	109.00
N3—C31—C32	115.7 (2)	C31—C32—H32A	109.00
O3—C31—N3	122.5 (2)	C31—C32—H32B	109.00
O3—C31—C32	121.8 (2)	C31—C32—H32C	109.00
C3—C4—H4	120.00	H32A—C32—H32B	109.00
C5—C4—H4	120.00	H32A—C32—H32C	109.00
C4—C5—H5	120.00	H32B—C32—H32C	110.00
			
C13—O12—C12—O11	−5.0 (4)	C31—N3—C3—C4	1.5 (4)
C13—O12—C12—C11	175.59 (19)	C3—N3—C31—O3	1.4 (4)
C12—O12—C13—C14	−177.9 (2)	C3—N3—C31—C32	−179.6 (2)
C12—O12—C13—C15	−60.6 (3)	O2—C2—C3—N3	−1.0 (3)
C12—O12—C13—C16	64.0 (3)	O2—C2—C3—C4	178.7 (2)
C6—N1—C2—O2	−180.0 (2)	N1—C2—C3—N3	178.89 (19)
C6—N1—C2—C3	0.2 (3)	N1—C2—C3—C4	−1.4 (3)
C11—N1—C2—O2	−4.9 (3)	N3—C3—C4—C5	−178.3 (2)
C11—N1—C2—C3	175.25 (19)	C2—C3—C4—C5	2.0 (4)
C2—N1—C6—C5	0.6 (4)	C3—C4—C5—C6	−1.3 (4)
C11—N1—C6—C5	−174.5 (2)	C4—C5—C6—N1	0.0 (4)
C2—N1—C11—C12	−81.1 (2)	N1—C11—C12—O11	11.0 (3)
C6—N1—C11—C12	94.2 (2)	N1—C11—C12—O12	−169.61 (18)
C31—N3—C3—C2	−178.8 (2)		

Symmetry codes: (i) −*x*+2, −*y*+1, −*z*; (ii) −*x*+2, *y*+1/2, −*z*+1/2; (iii) *x*+1, *y*, *z*; (iv) *x*, *y*+1, *z*; (v) −*x*+2, −*y*, −*z*; (vi) −*x*+2, *y*−1/2, −*z*+1/2; (vii) *x*, *y*−1, *z*; (viii) *x*−1, *y*, *z*.

### Hydrogen-bond geometry (Å, °)

**Table:**

*D*—H···*A*	*D*—H	H···*A*	*D*···*A*	*D*—H···*A*
N3—H3···O2^i^	0.8600	2.3400	3.164 (3)	161.00
C4—H4···O3	0.9500	2.2300	2.830 (3)	120.00
C14—H14A···O3^viii^	0.96	2.56	3.465 (4)	157
C15—H15A···O11	0.96	2.44	2.980 (4)	115
C16—H16A···O11	0.96	2.44	2.978 (5)	115
C32—H32C···O2^i^	0.96	2.33	3.222 (3)	155

Symmetry codes: (i) −*x*+2, −*y*+1, −*z*; (viii) *x*−1, *y*, *z*.
